# Supplementary material for: Learning induces the translin/trax RNase complex to express activin receptors for persistent memory
Source: eLife. 2017 Sep 20;6:e27872. doi: 10.7554/eLife.27872 (PMC5606845; doi:10.7554/eLife.27872)
Supplement: Figure 3—source data 1. — (Descending order of aggregate context scores). [file elife-27872-fig3-data1.docx]

**Figure 3–Table Supplement 1**. Common target genes of the conserved mRNA targets of let-7, miR-125 and miR-128. (Descending order of aggregate context scores)

| **RefSeq ID** | **Symbol** | **Name** | **Context Score (Mouse)** | | | **Aggregate** **context** **scores** |
| --- | --- | --- | --- | --- | --- | --- |
|  |  |  | **Let7c** | **miR 128** | **miR 125b** |  |
| NM_001111031 | ACVR1C | activin A receptor, type IC | -0.55 | -0.51 | -0.09 | -1.15 |
| NM_024674 | LIN28A | lin-28 homolog A (C. elegans) | -0.43 | -0.15 | -0.3 | -0.88 |
| NM_030915 | LBH | limb bud and heart development | -0.08 | -0.39 | -0.4 | -0.87 |
| NM_001134375 | CCNJ | cyclin J | -0.31 | -0.1 | -0.3 | -0.71 |
| NM_015177 | DTX4 | deltex 4, E3 ubiquitin ligase | -0.07 | -0.2 | -0.44 | -0.71 |
| NM_001099287 | NIPAL4 | NIPA-like domain containing 4 | -0.15 | -0.24 | -0.3 | -0.69 |
| NM_001145365 | ZNF652 | zinc finger protein 652 | > -0.08 | -0.29 | -0.11 | > -0.48 |
| NM_002657 | PLAGL2 | pleiomorphic adenoma gene-like 2 | -0.11 | -0.22 | -0.06 | -0.39 |
| NM_017759 | INO80D | INO80 complex subunit D | -0.1 | > -0.09 | -0.13 | > -0.32 |
| NM_001178137 | CPEB3 | cytoplasmic polyadenylation element binding protein 3 | -0.12 | > -0.02 | -0.18 | > -0.32 |
| NM_001170765 | LCOR | ligand dependent nuclear receptor corepressor | -0.13 | -0.08 | -0.1 | -0.31 |
| NM_020335 | VANGL2 | VANGL planar cell polarity protein 2 | > -0.03 | -0.09 | -0.07 | > -0.19 |
| NM_001198783 | POU2F1 | POU class 2 homeobox 1 | > -0.02 | -0.13 | -0.03 | > -0.18 |
